# Gene-Microbiome Co-expression Networks in Colon Cancer

**DOI:** 10.3389/fgene.2021.617505

**Published:** 2021-02-15

**Authors:** Irving Uriarte-Navarrete, Enrique Hernández-Lemus, Guillermo de Anda-Jáuregui

**Affiliations:** ^1^Computational Genomics Division, National Institute of Genomic Medicine, Mexico City, Mexico; ^2^Centro de Ciencias de la Complejidad, Universidad Nacional Autónoma de México, Mexico City, Mexico; ^3^Conacyt Research Chairs, National Council on Science and Technology, Mexico City, Mexico

**Keywords:** colorectal cancer, microbiome, tumor progression, probabilistic multilayer networks, information theory

## Abstract

It is known that cancer onset and development arise from complex, multi-factorial phenomena spanning from the molecular, functional, micro-environmental, and cellular up to the tissular and organismal levels. Important advances have been made in the systematic analysis of the molecular (mostly genomic and transcriptomic) within large studies of high throughput data such as The Cancer Genome Atlas collaboration. However, the role of the microbiome in the induction of biological changes needed to reach these pathological states remains to be explored, largely because of scarce experimental data. In recent work a non-standard bioinformatics strategy was used to indirectly quantify microbial abundance from TCGA RNA-seq data, allowing the evaluation of the microbiome in well-characterized cancer patients, thus opening the way to studies incorporating the molecular and microbiome dimensions altogether. In this work, we used such recently described approaches for the quantification of microbial species alongside with gene expression. With this, we will reconstruct bipartite networks linking microbial abundance and gene expression in the context of colon cancer, by resorting to network reconstruction based on measures from information theory. The rationale is that microbial communities may induce biological changes important for the cancerous state. We analyzed changes in microbiome-gene interactions in the context of early (stages I and II) and late (stages III and IV) colon cancer, studied changes in network descriptors, and identify key discriminating features for early and late stage colon cancer. We found that early stage bipartite network is associated with the establishment of structural features in the tumor cells, whereas late stage is related to more advance signaling and metabolic features. This functional divergence thus arise as a consequence of changes in the organization of the corresponding gene-microorganism co-expression networks.

## Introduction

Colon cancer is consistently ranked among the top five contributors to cancer deaths worldwide (Bray et al., 2018). Its incidence and mortality are rapidly rising in developing countries, possibly influenced by changes in lifestyle and socioeconomic conditions. It is expected that this trend will actually further increase according to recent studies (Arnold et al., 2017).

As with many other cancers, colon cancer is known to have a genetic component as well as environmental factors which further modulate or increase the risks. Its molecular determinants include genomic, regulatory, and epigenomic components (Raskov et al., 2020) whereas the environmental component is also multifactorial, ranging from toxicological exposure (Fernández-Martínez et al., 2020), physical activity (Friedenreich et al., 2020), dietary habits and more. A more recent factor that is an important research topic is the role that microbiome interactions may be playing at the molecular and patho-physiological levels.

Recent findings have pointed out to different, sometimes disparate phenomena, such as the influence of bacterial protein toxins (Fiorentini et al., 2020), altered microbiome composition (Xu et al., 2020), and the non-rational use of antibiotics (Simin et al., 2020). Among these, microbome-host interactions are hypothesized to modulate and integrate these diverse signals (Yang et al., 2020). For instance, experimental evidence has been found for functional alterations mediated by microorganisms involved in colon cancer progression (Yu et al., 2020). It is currently accepted that these complex biomolecular and organismal interactions can be better understood using a systems biology approach (Peñalver Bernabé et al., 2018).

In the context of oncology, network biology has proven to be a powerful tool for the integration of multiple high throughput technologies (de Anda-Jáuregui and Hernández-Lemus, 2020). Networks provide flexible frameworks to represent the relevant physio-pathological interactions present in the tumor environments. For instance, bipartite networks have been used to represent gene expression control by micro-RNAs; a strategy that allows not only to describe statistical associations, but also to identify putative functional associations (de Anda-Jáuregui et al., 2018, 2019).

In this work, we reconstructed bipartite networks that capture the statistical dependence between microorganism abundance and gene expression in *early* (stages I and II) and *late* (stages III and IV) colon cancer, using data from The Cancer Genome Atlas (TCGA). We analyze these networks to identify changes in the relative relevance of microorganisms between these conditions, in terms of their topological role in their respective networks. We analyzed genes associated to the highest ranked microorganisms in each network as a means to identify changes in associated biological functions. This work hence aims to provide novel insights into microorganism-mediated functional alterations potentially involved in colon cancer progression.

## Materials and Methods

For this work, we collected gene expression data from TCGA, along with microorganism quantification data that was generated by Poore et al. (2020), for the same 269 samples. We classified these samples into *early* (*n* = 150) and *late* (*n* = 119) colon cancer based on tumor stages as provided by TCGA metadata.

**Figure 1:**
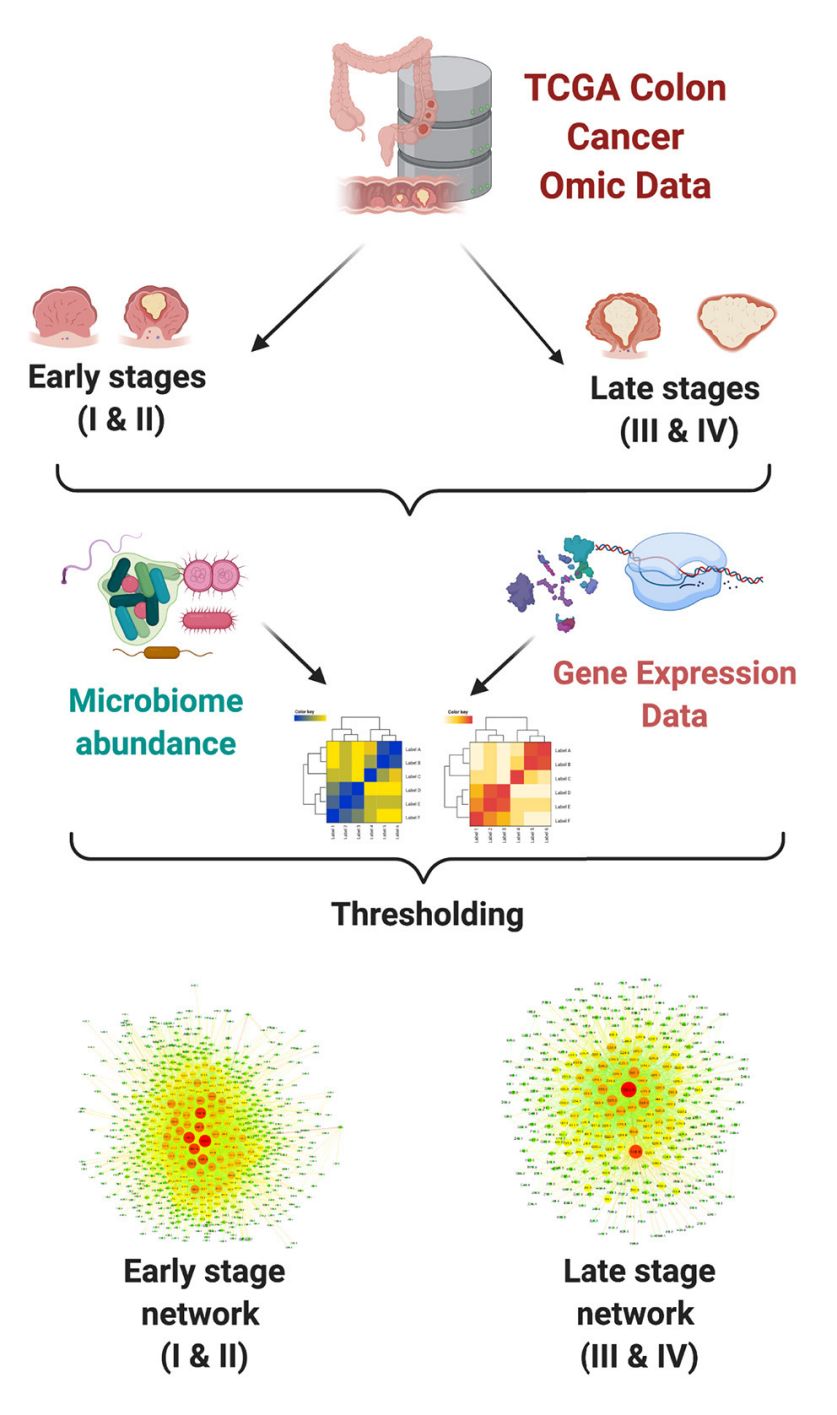
Network analysis pipeline.

Interactions between each pair of measured microorganism and gene were detected using mutual information (MI) as a measure for statistical dependence. The highest ranked interactions were kept in order to reconstruct bipartite networks for each group. Downstream analyses included topological characterization and functional enrichment analysis. In Figure 1, we present a schematic representation of our analysis pipeline.

### Gene Expression Data

We used data from TCGA, obtained through the Genome Data Commons portal. We used level three pre-processed gene expression data; the full analysis pipeline is documented at https://docs.gdc.cancer.gov/Data/Bioinformatics_Pipelines/Expression_mRNA_Pipeline/; briefly, RNA-seq data is aligned using *STAR* (Dobin et al., 2012), and reads mapped to each gene are counted using *HT-SEQ* (Anders et al., 2014); Read counts are normalized using the Fragments per Kilobase of transcript per Million mapped reads (FPKM) calculation, which divides counts by the gene length and the total number of reads mapped to protein-coding genes.

Based on the available metadata, samples with tumor stages I and II were grouped as early colon cancer, while samples with tumor stages III and IV were grouped as late colon cancer. Due to some samples being discarded from the microbiome quantification pipeline by the original authors (Poore et al., 2020, see next section), we ended up using 137 early stage and 64 late stage samples (see Supplementary File 1 for the TCGA identificators of the used samples).

### Microorganism Abundance Data

We used the public dataset generated in Poore et al. (2020) as our source for microorganism abundance data. Briefly, in said work the authors were able to quantify microorganism abundance in TCGA tumor samples via a novel bioinformatics approach. Briefly, They took raw whole genome sequencing (WGS) data and analyzed the nearly 0.9% of total sequencing reads were classified as non-human and assigned to bacteria, archaea, or viruses at the genus level using *Kraken* (Wood and Salzberg, 2014); which matches k-mers to taxa in a reference database. Normalization was performed considering sample number within a cancer type and sample type. To correct for batch effects, discrete taxonomical counts are converted to log-counts per million per sample using *Voom* (Law et al., 2014), and a secondary supervised normalization was performed to remove significant batch effects. Additionally, contamination concerns were addressed using the Bayesian source tracking model SourceTracker2 (Knights et al., 2011). Based on their quantification, we crossed microorganism abundance and gene expression data at the aliquot level, to ensure biological comparability between the datasets.

### Microbiome-Gene Co-expression Quantification

Having matched gene expression and microorganism abundance data organized into *expression matrices*, we calculated mutual information for each pair of *microorganism* × *gene*. Mutual information is the maximum likelihood information theoretic measure of statistical dependence. Since it is capable to capture non-linear relationships between features, it has been successfully used for gene co-expression network reconstruction (de Anda-Jáuregui et al., 2016; He et al., 2017). It has also been previously used for bipartite network reconstruction of multiomic data (de Anda-Jáuregui et al., 2018, 2019). In this work, we calculated MI using the infotheo package in R.

Once MI values were calculated, we selected those interactions above the 99.5 quantile to be considered as links on a bipartite network: B(microorganism,gene); A bipartite graph (or bigraph) is a network whose nodes can be divided into two disjoint sets U and V such that each link connects a U-node to a V-node. Importantly, no links are found between two nodes belonging to the same set (Barabási et al., 2016). For mutual information calculation, data is discretized using the equal frequency method (Meyer 2008), which assigns each observation to one of N bins, with N being the number of observations. The discretized vectors are then used as the input for proper mutual information calculation, using an entropy estimation of the empirical probability distribution. Both of these calculations were performed using the infotheo package for R.

For completeness, the reconstructed networks contained all measured microorganisms (*N* = 4, 450) and protein-coding genes (*N* = 16, 593), even if they do not participate in any link (that is, they have connectivity degree *k* = 0). The threshold was selected based on previous analyses of multi-omic bipartite networks (de Anda-Jáuregui et al., 2018, 2019); we must acknowledge that by using this threshold we guarantee fair comparisons between the reconstructed networks; however, the structure and composition of these networks will not be comparable to networks generated through other methods (including the selection of a different threshold).

### Network Analyses

We characterized the topology of each of the generated using a combination of the igraph (Csardi and Nepusz, 2006) in R and networkx (Hagberg et al., 2008) in Python. Additionally, we used Cytoscape (Shannon, 2003) to generate network visualizations. In this work, we focused mainly on centrality measures including degree, bipartite clustering coefficient, and redundancy coefficients (Latapy et al., 2008). Comparisons between appropriate distributions were evaluated using the Kolmogorov-Smirnov test.

### Functional Enrichment of High-Degree Microorganism Gene Neighborhoods

**Figure 2:**
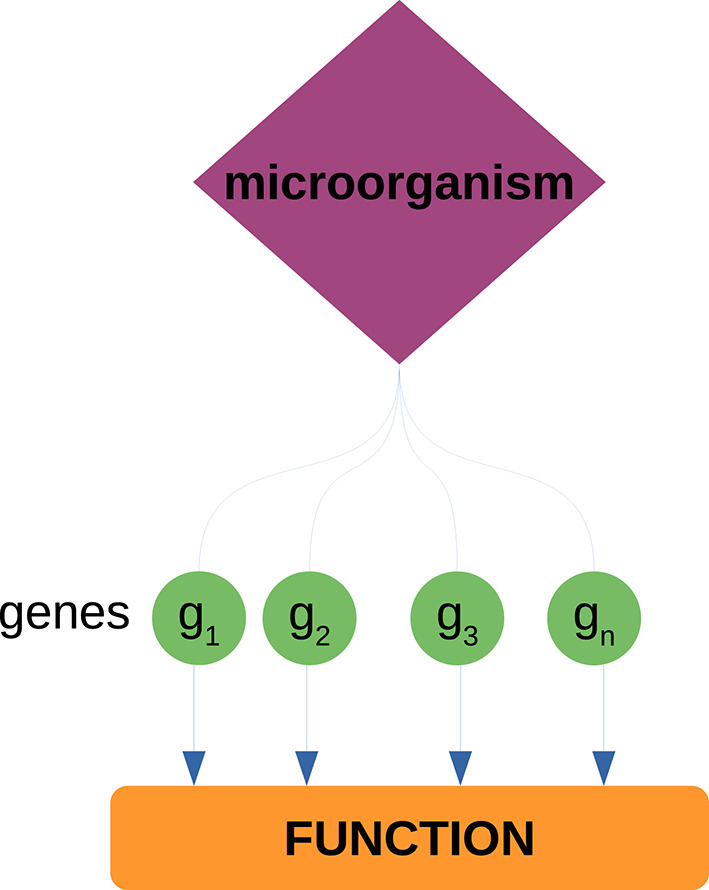
Enrichment of host biological functions associated to microorganisms through their gene neighborhoods. Each microorganism has a set of neighbor genes in the bipartite network. This gene set is tested against a set of known biological functions (as annotated in the GO database) through the hypergeometric test. Through these procedure, we can associate known biological functions from the host to each of the measured microorganisms.

We analyzed the neighborhoods of the highest ranked microorganisms (based on their degree) to identify host biological functions associated to these microorganisms. To do so, we performed over-representation analysis (ORA) via FDR-corrected hypergeometric tests for biological processes and molecular functions (as annotated in the Gene Ontology database) using the WebgestaltR (Liao et al., 2019) package. Parameters for ORA considered the full genome as the reference set, and a false discovery rate (FDR) threshold of 0.05. It should be noted that the enrichment is performed over the set of *genes* that conform the *neighborhood* of each *microorganism*; this is to identify biological functions from the host that can be associated to microorganisms through their co-expressed genes (see Figure 2). We further used natural language processing tools from the tm package in R (Meyer et al., 2008) to compare identified functions and processes, by tokenizing their names and descriptions and identifying the most mentioned keywords or tokens.

## Results and Discussion

### Microorganism-Gene Co-expression Networks Are Topologically Similar in Early and Late Colon Cancer

**Figure 3:**
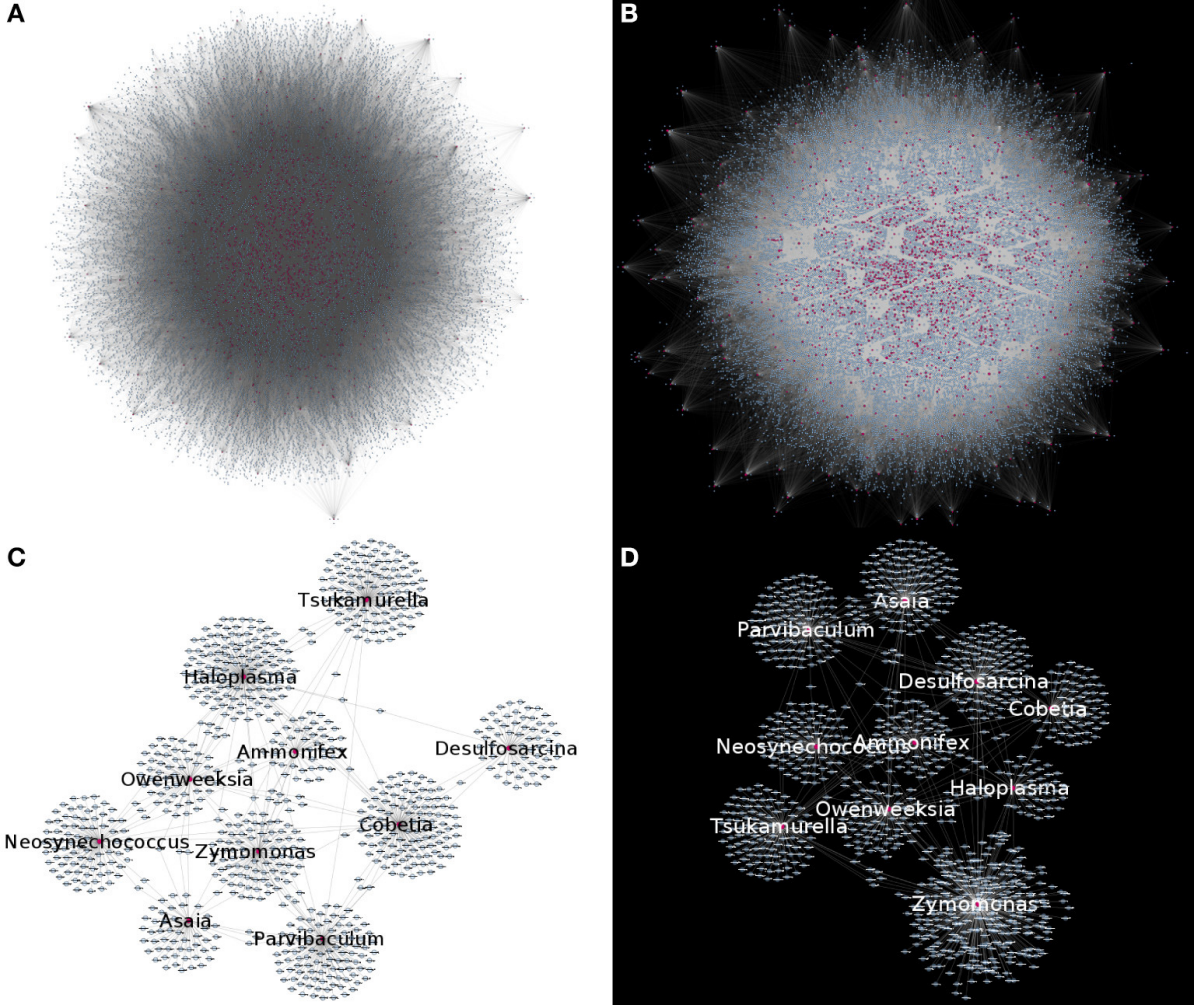
Microorganism-gene co-expression networks for early **(A)** and late **(B)** colon cancer. In this visualizations, microorganisms are colored purple and genes are colored blue. Nodes with degree *k* = 0 are removed for visualization purposes, highlighting that in both networks, connected nodes form a single giant component. **(C,D)** Show a subset of the early **(C)** or late **(D)** networks, highlighting the most connected microorganisms.

By studying bipartite networks, we wanted to know what are some possible ways in which the presence of microorganisms may affect the host's response (as proxied by changes in gene expression highly correlated with microbial abundance) and vice versa. Clues to this may be provided by the microbe-gene links. The reconstructed microorganism-gene co-expression networks for the early and late stages of colon cancer exhibit a similar global topology. They are both dominated by a giant connected component that contains all detected links. This giant connected component is composed of all measured microorganisms, and over 80% of measured genes. It should be noted that in the case of both genes and microorganisms, presence in the network is not directly correlated by the abundance in the original measurements, nor biased due to zero-inflation effects (see Supplementary File 2). Figure 3 depicts these networks. Table 1 presents the global topological features of these networks.

**Table 1:** General network descriptors.

	**Early**	**Late**
Genes (*k*>0)	16,593	17,535
Microorganisms (*k*>0)	1,464	
Edges	143,320	143,321
Giant connected component?	Yes	
GCC^**^ size	18,057	18,999
GCC^**^ node similarity^*^	91.79%	
Edge similarity^*^	0.28%	

* *Similarity expressed as percentualized Jaccard index*.

** *GCC, giant connected component*.

**Figure 4:**
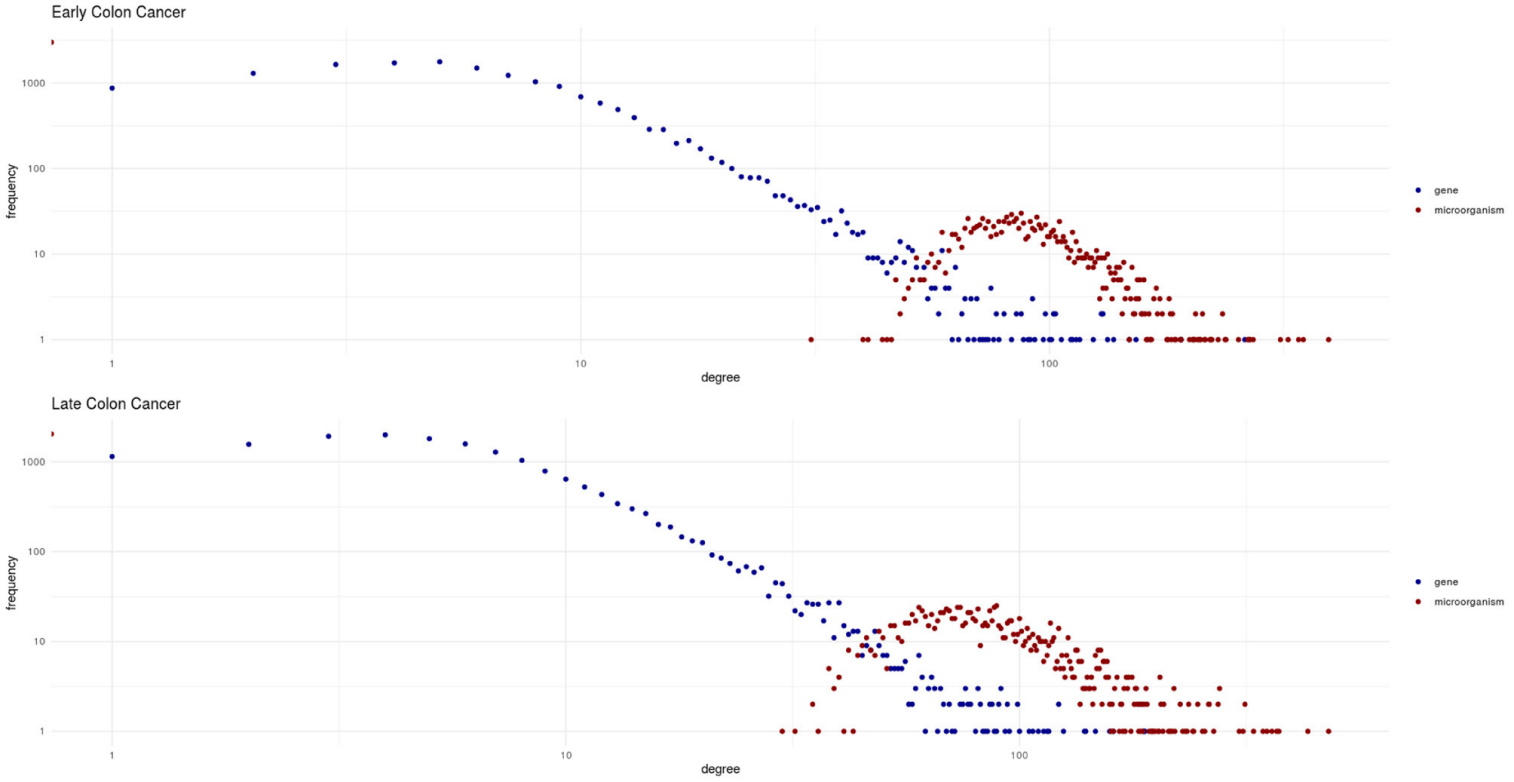
Degree distributions for the early and late colon cancer networks. Values for microorganisms are shown in red, and values for genes are shown in blue. Notice how genes exhibit a heavy-tailed distribution, whereas a different behavior is observed for microorganisms in both networks.

The bipartite degree distributions of these networks (seen in Figure 4) are quite similar between early and late stage. In this context, it is more informative to assess the degree distributions for each type of nodes (microorganisms and genes) separately. In this regard, we observe that in both networks, genes follow a heavy-tailed distribution (blue dots in Figure 4); that is, most genes are connected to few microorganisms, whereas a few genes are connected to many microorganisms. Meanwhile, microorganism nodes (red dots in Figure 4) exhibit a different pattern: a curve with no low-degree nodes; indicating that every detected microorganism has putative effects on the expression of a relatively large set of genes. In any case, the distributions for both genes and microorganisms are similar between early and late stages cancer networks.

**Figure 5:**
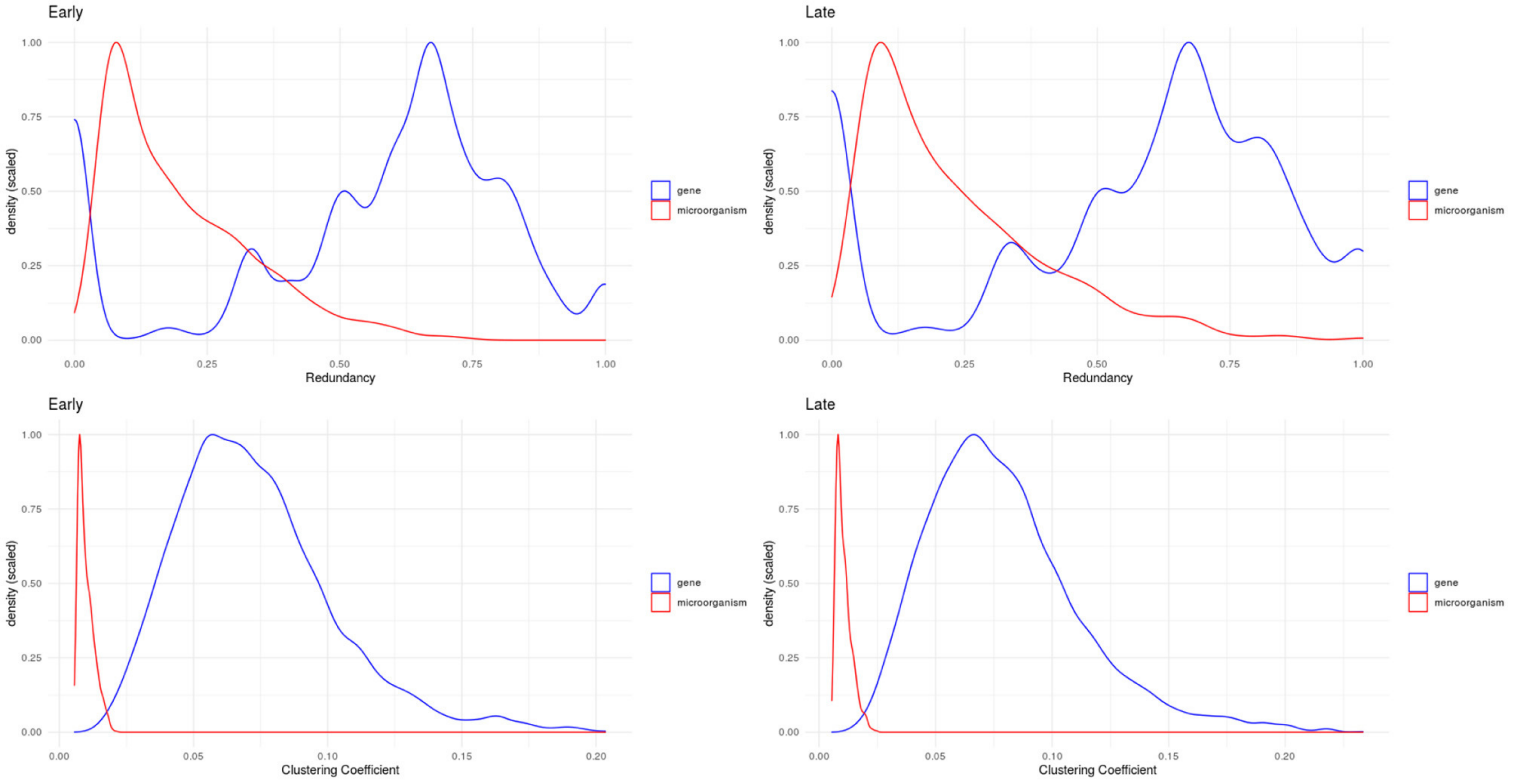
Density plots for redundancy **(top)** and clustering **(bottom)** coefficients. We can see how microorganisms (red lines) are significantly less redundant and clustered than genes within these networks.

We evaluated two other topological properties of the nodes in these networks: the clustering and redundancy coefficients (see Figure 5).

Network redundancy (sometimes called path degeneracy) is related to how many different paths or trajectories can be taken to go from one node to another. Unlike trees or loosely connected networks, complex networks (such as the ones discussed here) are characterized by being highly redundant. This means that there are multiple (sometimes many) different paths connecting two given nodes. For probabilistic networks this implies that the Markov blanket (the subset of the network with the useful connectivity information) spans much of the network. This in turn implies that to break up (percolate, in technical terms) the network to pieces, one must remove a large number of links. In the case of bipartite networks, the concept of redundancy has to be adapted, since neighborhood overlaps correspond to links obtained in several ways during projection which are not distinguishable. Then redundancy is caused by nodes that when removed from the bipartite graph, do not cause significant changes in the projection (Latapy et al., 2008).

The clustering coefficient is a quantitative measure of the tendency of nodes in a graph to cluster together. It is calculated for a node (local clustering coefficient), as the ratio of the number of “triangles” (technically “closed triplets”) formed by links connected to this node, to all possible triangles that can be formed with this node and its immediate neighbors. The global clustering coefficient is a network quantity, which is indeed the average of the local clustering coefficient of all the nodes in connected components of the network. In the case of clustering coefficients for bipartite networks, these measure the probability that given four nodes with three links, they are actually all connected with four links (all the possible links in a bipartite configuration of four nodes) (Latapy et al., 2008).

In bipartite networks, these are measures of the contribution of a given node to the connectivity of nodes of the opposite type (Latapy et al., 2008). We observe that in the case of microorganisms (red curves in Figure 5), these exhibit low values: this indicates that there is no single microorganism through which most genes could interact. Meanwhile, genes (blue curves in Figure 5) exhibit higher values, meaning that gene-mediated connections between microorganisms are, on average, more likely to be redundant. Table 2 shows the statistical differences between the evaluated distributions.

**Table 2:** Distribution comparison (Kolmogorov-Smirnov test).

***p*-value, KS-test**	**Redundancy**	**Clustering coefficient**
Microorganisms	6.037e-05	0.01244
Genes	0.01017	0.01838

Despite these overall similarities, networks for early and late colon cancer exhibit notable differences in terms of their connections. Although the composition of the GCC is fundamentally similar in terms of the microorganisms and genes found in it, the way in which this are connected is completely dissimilar, with a Jaccard similarity for edges of only 0.28%.

This differences in connectivity in turn explain the different degree ranking of both microorganisms and genes. The ranked list of microorganisms and genes show poor correlation between the early and late stages (Spearman ρ of 0.015 for microorganisms and 0.269 for genes). Due to these differences, the highest ranked microorganisms are (a) different in the early and late stages of colon cancer and (b) have a different set of associated genes. With this in mind, we explored how these facts change the set of host biological functions associated to the most connected microorganisms.

Regarding microorganisms, Tables 3, 4 present the top 10 highly connected microorganism (at the genus level) in the gene microorganism bipartite networks for early and late stage colon cancer, respectively.

**Table 3:** Early colon cancer: top 10 highest ranked microorganism by degree.

**Genus**	**Connectivity degree**
Ilumatobacter	394
Rhodospirillum	348
Nitrosospira	340
Pontibacter	323
Shinella	311
Phaseolibacter	272
Vogesella	268
Azospirillum	267
Rubrivivax	265
Thermodesulfovibrio	253

**Table 4:** Late colon cancer: top 10 highest ranked microorganism by degree.

**Genus**	**Connectivity degree**
Desulfurella	480
Nitriliruptor	432
Jeotgalicoccus	373
Actinocatenispora	369
Cryocola	360
Dactylosporangium	351
Pelomonas	344
Rhodovulum	328
Zymomonas	314
Methylomonas	314

By examining Tables 3, 4, it may be surprising that most of the microbial species themselves have not been reported to be related with the onset and development of colon cancer. This of course may be explained by the fact that systematic high-throughput studies of the relationship between cancer and microbial dysbiosis are indeed still being developed. So the absence of evidence may not (yet) be taken as evidence of absence. However, in the next subsection we will see how, even though the organisms themselves may not sound that familiar, the statistically dependent gene neighborhoods of such microorganisms will recapitulate relevant functional features known in the biology of colon cancer.

### Host Biological Functions Associated to Highly Connected Microorganisms Change With Colon Cancer Progression

We set to identify functions that could be linked to microorganisms detected in the early and late stage tumors. Since there is no annotation of human biological functions associated to microorganisms, we performed ORA on the gene neighborhoods of the top 10 highest ranked microorganisms by degree, searching for enrichment of biological processes and molecular functions annotated in Gene Ontology.

#### Enrichment Results for Biological Processes

The biological processes branch of the Gene Ontology is devoted to biologically relevant functional processes, some of these have clearly understood biomolecular mechanisms and some others are yet to be fully dissected. However, they allow for an advancement in our understanding of the molecular and cellular physiology behind gene and protein interactions.

Statistically enriched biological processes may represent functional processes in which the host-microbiome interactions are manifested. As we will see, some of these actually correspond to well-known hallmarks of cancer.

**Figure 6:**
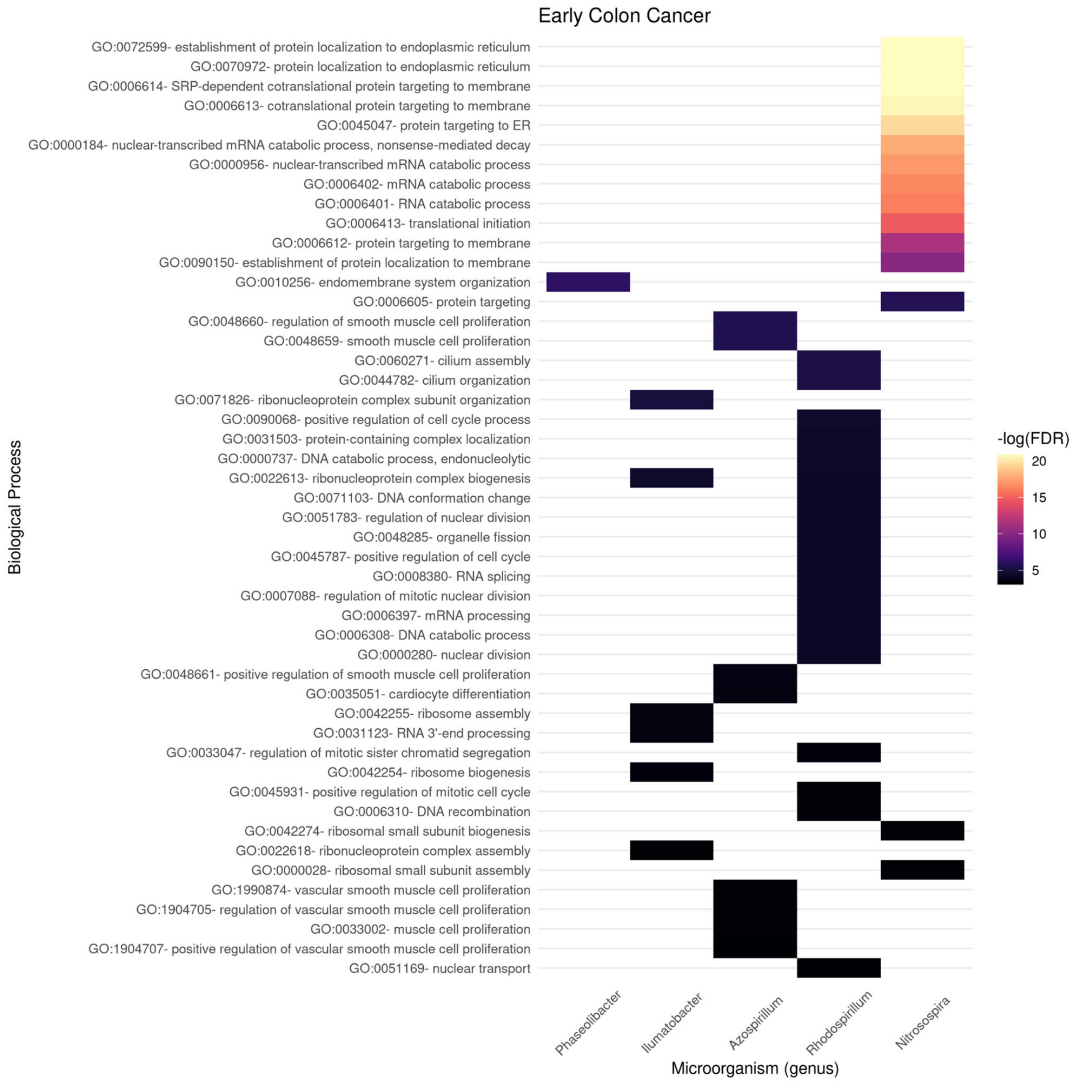
Functional enrichment of highly connected microorganisms in the early colon cancer network—biological processes.

**Figure 7:**
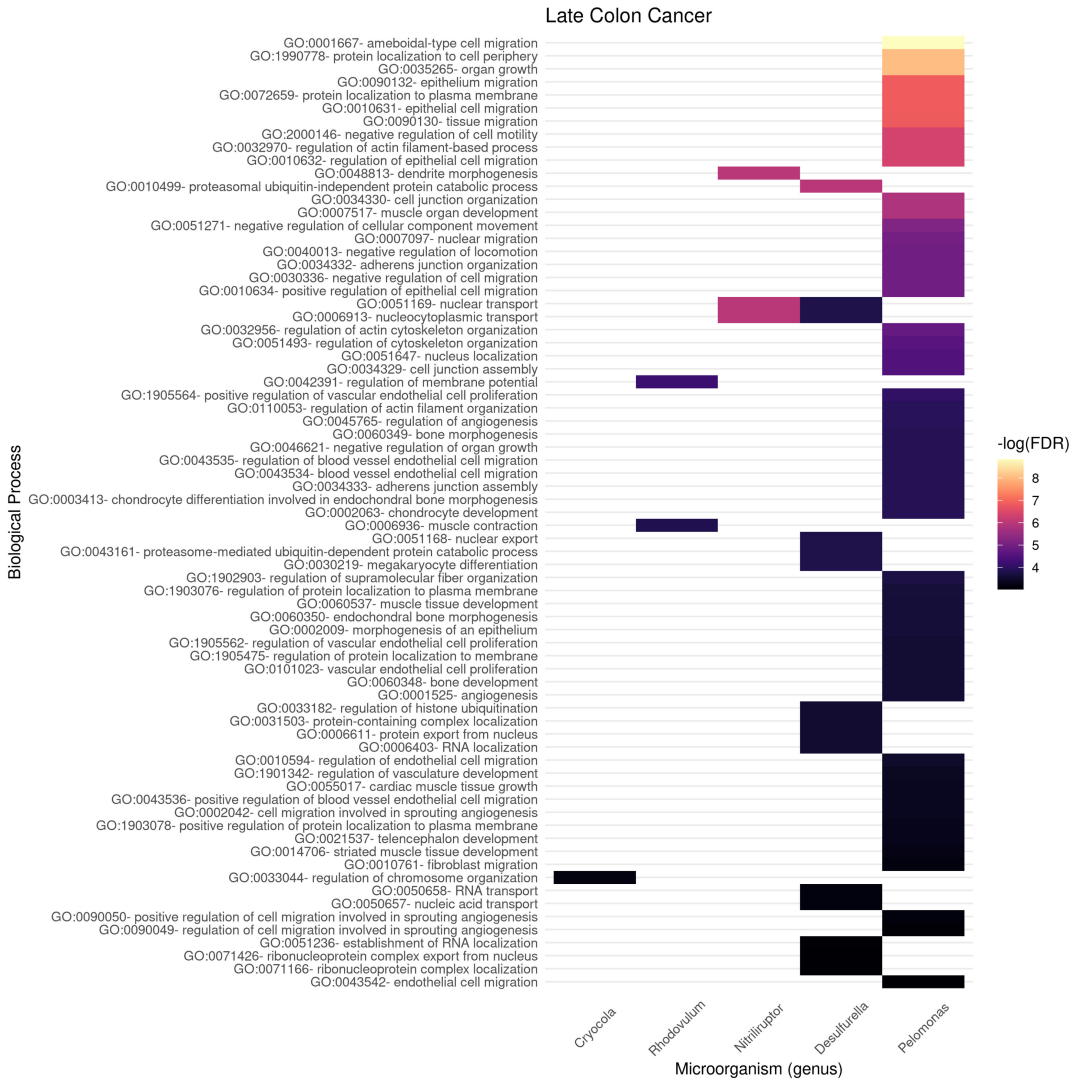
Functional enrichment of highly connected microorganisms in the late colon cancer network—biological processes.

In Figures 6, 7, we present the results of these enrichment analyses as a heatmap. Notice that even if we performed the analyses for the 10 highest ranked microorganisms, only five genus were significantly associated to functions through their gene neighborhoods in each network.

Notably, higher enrichment values (in terms of FDR) are found in the early stage (Figure 6) than in the late stage (Figure 7). The interpretation is that biological functions are perhaps better mapped to the gene neighborhoods in the early colon cancer network—possibly indicating a more coordinated response to these microorganisms.

We identified only two biological processes appearing both in the early and late networks. These are *protein-containing complex localization* and *nuclear transport*. To better understand the functional differences identified, we tokenized the names of the detected biological processes and compared them between the early and late networks.

**Figure 8:**
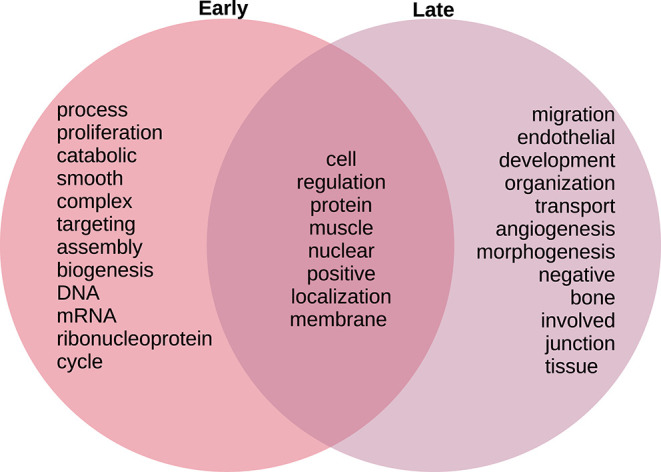
Venn diagram of top 20 most mentioned concepts in biological processes associated to early and late colon cancer.

In Figure 8, we compare and contrast the terms associated to these biological processes. We observe in the early stages concepts associated to tumorigenesis such as *proliferation, biogenesis*, and (cell) *cycle*; as well as nucleic acids. Meanwhile, in the late stages, we observe terms that could be associated to late-stage cancer such as *migration* and *angiogenesis*. Concepts shared between both stages include *regulation, muscle*, and *protein*. For the full set of enrichment results, please refer to Supplementary File 3.

#### Enrichment Results for Molecular Functions

By recognizing that our understanding of the way microbiome-host interactions may be playing a role on the onset and development of cancer-associated biological processes is still quite incipient, we decided to also examine the molecular functions dimension of the Gene Ontology. This is so since molecular function refers to specific chemical and biochemical interactions of a more general nature that may be related to one, or more commonly to a large set of biological processes.

The rationale is that molecular species related to the entangled multi-microbial metabolism are possible interacting with the molecules involved in human (and in particular tumor and tumor micro-environment) cells.

**Figure 9:**
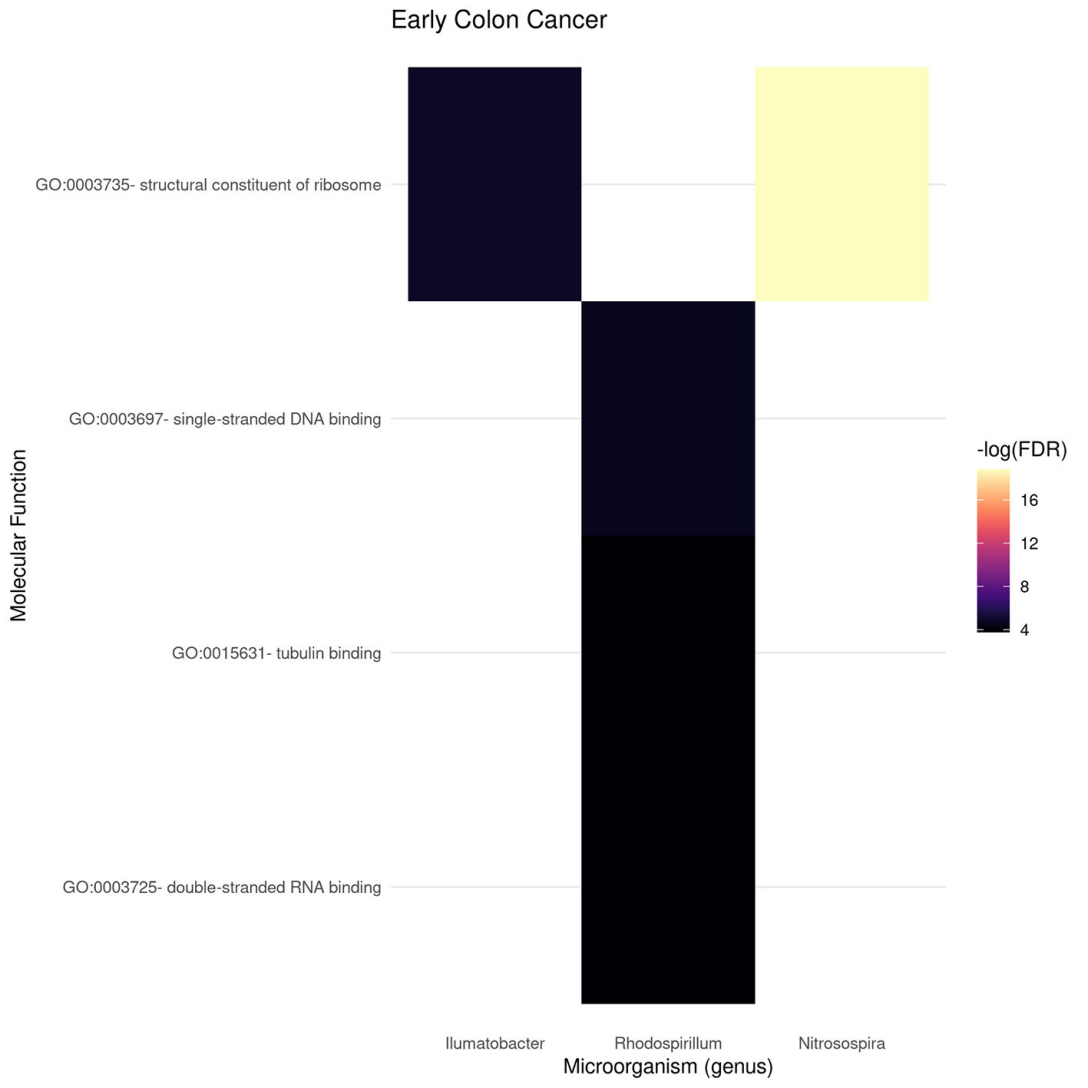
Functional enrichment of highly connected microorganisms in the early colon cancer network—molecular functions.

**Figure 10:**
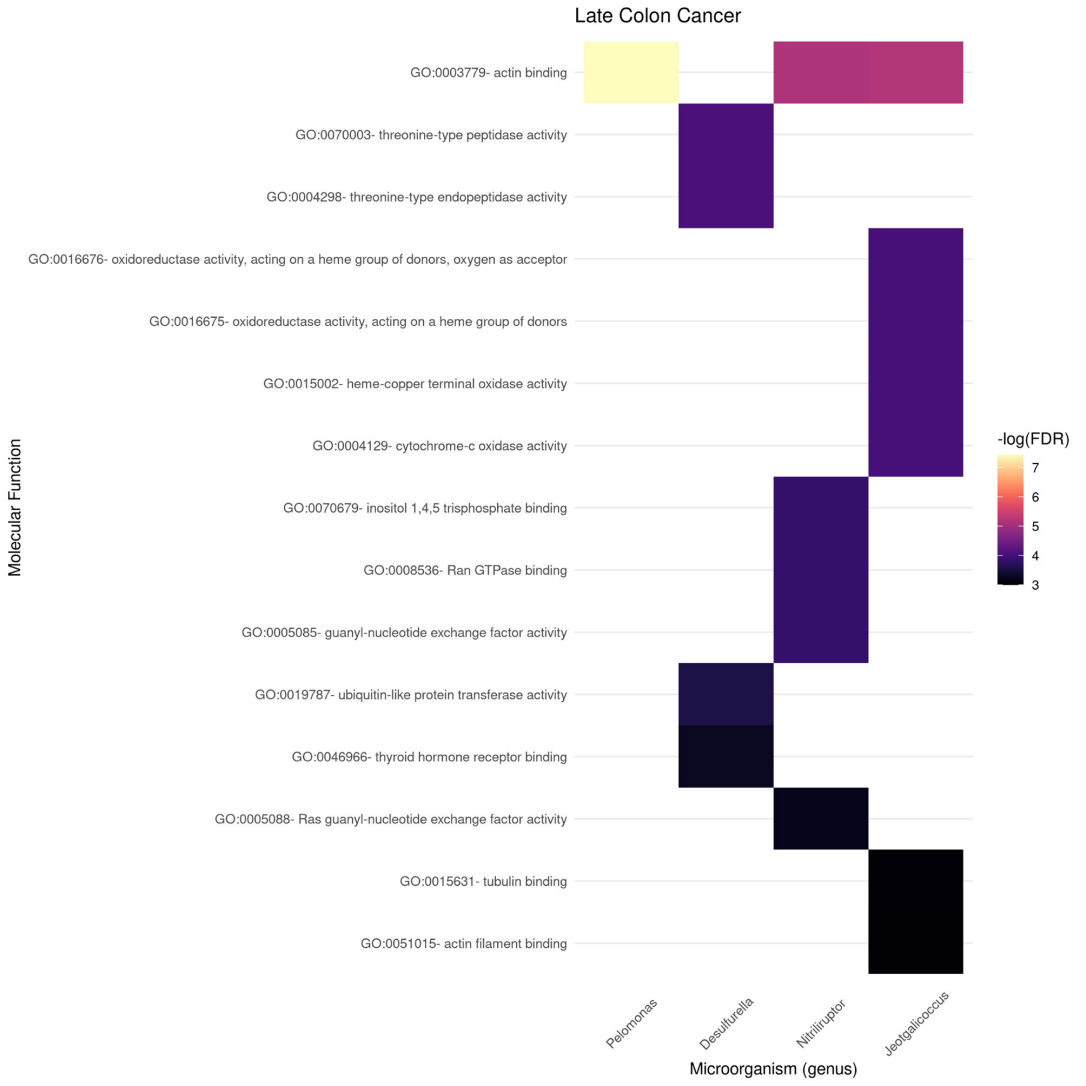
Functional enrichment of highly connected microorganisms in the late colon cancer network—molecular functions.

Figures 9, 10 present the molecular function enrichment analysis for the early and late colon cancer networks. As in the case of biological processes, molecular functions are enriched on *different* microbial genus in the early and late stage networks. It is worth noticing that the more significant physico-chemical functions in the early stage correspond to structural features (particularly enriched for the gene-neighborhood of the Nitrosospira genus, see Figure 9) whereas the more enriched molecular functions in the late stage network corresponds to actin binding for genes in the network vicinity of the Pelomonas genus (Figure 10).

We can also notice in Figure 10 that other microbial genuses' gene neighborhoods are highly enriched for molecular functions, such is the case of Jeotgallicoccus for actin binding, and to several types of oxido-reductase, as well as cytochrome-oxidase activity; and the case of Nitriliruptor for GTP-ase and nucleotide binding, and Desulphurella for ubiquitin and thyroid receptor activity.

**Figure 11:**
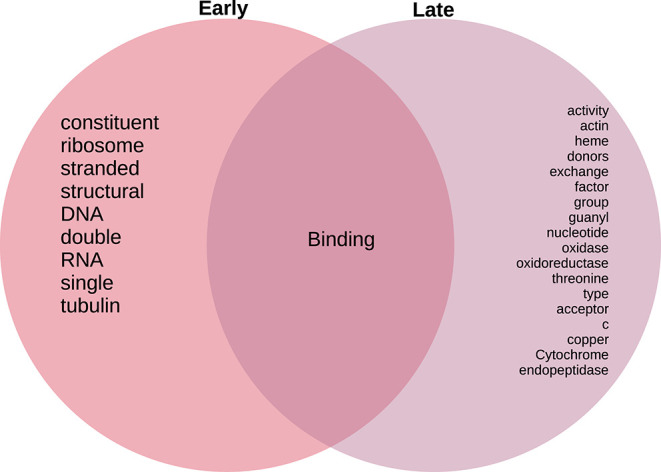
Venn diagram of top 20 most mentioned concepts in molecular functions associated to early and late colon cancer.

As in the case of the Biological Processes enrichment analysis, Figure 11 presents the results of natural language processing and tokenization of terms resulting in the statistically significant enrichment GO-categories. As it was mentioned, early stage molecular functions are somehow related to structural cellular features, whereas late stage are related to cellular metabolism and transport processes, being binding phenomena the common function at the intersection of both stage networks. For the full set of enrichment results, please refer to Supplementary File 3.

## Discussion

### Topology of the Microbiome-Gene Co-expression Networks

Complex networks are characterized by their composition and global topological structure, that is by what are their elements and how are these connected in the networks. As presented in Figures 2–4 and Table 1 in results, the global topological structures of early and late stage colorectal cancer bipartite networks are indeed quite similar. Approximately equal sizes in terms of number of nodes and edges. Similar size of their giant connected components and even a very high value of node similarity in their GCCs. However, as it can be seen in Table 1 the edge similarity (a quantity proportional to the number of shared edges between the two networks) is actually extremely small (0.28%). This means that even if the elementary components of the networks (i.e., the genes and microorganisms) are almost the same and the global network features are so similar, the actual networks are indeed quite different, something unsurprising given that they represent two different biological scenarios.

Also noteworthy is the fact that by examining Figure 4 we could notice that the two different types of nodes (genes and microorganisms) present striking differences in their degree connectivity probability distributions (blue dots representing genes and red dots microorganisms) and that the same patterns is observed for early and late stage colorectal cancer. The degree distributions for genes present long-tailed distributions that have been thoroughly characterized in complex biomolecular networks. In those long-tailed distributions one can notice how most genes have a relatively low number of connections whereas a few hub genes are densely connected in the networks.

Microorganisms, on the other hand present a rather different degree distribution scenario. In both networks, microorganisms show a more symmetric short-tailed distribution in which a most microorganisms are highly connected and present narrower variability in their connectivity degree. This difference perhaps represent that microbial communities somehow serve as integrating entities in the bipartite network. This, in turn, may be related with the low redundancy coefficients displayed by microorganisms in both networks as it can be seen in Figure 5 (top row). Low redundancy of the specific microbial agents may prove later to have relevance for the design of microbiome-driven therapeutic strategies, though it is still very early to further speculate on this.

One relevant and complementary aspect to consider on the role that gene-microbial interactions may play can be glimpsed by looking at the probability density distributions for the clustering coefficient (Figure 5 bottom row). We can see that in both networks (early and late stage) microorganisms present low values of clustering coefficient, whereas for genes there are wider probability distributions. Microorganisms are highly connected but not so-clustered. This in turn contributes to their being less redundant. This also may imply that the gene-microbiome co-expression program in the cancer networks is shaped by the full set of gene-microbial interactions and is not dominated by a few central players. This fact has been already discussed in the literature: physio-pathological phenomena related to microbial activity is, in general, influenced by microbiome dysbiosis rather than by the activity of a single or a few microorganisms.

### Changes in Network Composition and Relative Importance

The latter points led us to discuss on how, even if the whole set of microorganisms is present in both, early and late stage colorectal cancer networks, their connectivity and importance in information processing within the networks vastly differ.

Consider Tables 3, 4, for instance. There, we can see that the top 10 highly ranked microorganisms (that is, those with higher statistical dependencies and connectivity in the gene-microbial co-expression networks) are quite different. Indeed, no microorganism is present simultaneously at the top 10 of both networks, even at the, somewhat general, genus level presented here. This points out to a possible *reprogramming* of the gene-microbiome regulatory structure associated with the phenotypic differences between early and late stage colorectal cancer.

Regarding the highest ranked microorganisms associated to early stage colon cancer (Table 3), we have found that, in the case of Rhodospirillum, for instance, it is known to be able to produce molecules such as L-asparaginase which is a regulator of telomerase activity that has been found able to act on human cancer and immune cells (Zhdanov et al., 2017a,b; Plyasova et al., 2020). Nitrosospira is associated with processes related to ammonia oxidation (Kowalchuk and Stephen, 2001) in connection with colon cancer (Bingham et al., 1996; Bruce et al., 2000; Davis and Milner, 2009; O'keefe, 2016). Pontibacter has been found enriched in patients with gastric cancer and correlated with TNM severity (Dong et al., 2019).

In the case of Shinella, significant abundance has been found in mucosal associated microbiota in patients with severe irritable bowel syndrome (Li et al., 2018), and also is known to be involved in the production of N-nitrosonornicotine, a strong (group 1) carcinogen (Qiu et al., 2016). Vogesella dysbiosis has been recently found associated with gastric cancer (Coker et al., 2018; Rajilic-Stojanovic et al., 2020), as well as with changes in the endometrial microbiota associated with inflammatory cytokines in endometrial cancer (Lu et al., 2020), and with esophageal squamous cell carcinoma (Lv et al., 2020).

As regards to Rubrivivax, it is able to produce a molecule rubrivivaxin that is a cytotoxic agent and a COX-1 inhibitor (Kumavath et al., 2011). As is known COX-1 and COX-2 are relevant players in human colorectal cancer (Sano et al., 1995; Sinicrope and Gill, 2004; Pannunzio and Coluccia, 2018). Rubrivivax dysbiosis has also been found present in connection to lung cancer (Greathouse et al., 2018).

Thermodesulfovibrio has been recently discussed to play a role in the modulation of FOXP3 and IL-17 involved in immune tolerance in colon cancer (Bergsten et al., 2020). Sulfate reducing bacteria, also including Desulphurella are known to be associated with the pathogenesis of colorectal cancer (Kováč et al., 2017; Suri et al., 2019). Nitriliruptor has been reported to be involved colorectal cancer (Marzban et al., 2020), its dysbiosis has been mentioned also in connection to renal carcinomas (Wang et al., 2020) and severe cases of irritable bowel syndrome (Zhuang et al., 2018).

In connection with microorganisms associated with late stage colon cancer (Table 4), Jeotgalicoccus abundance has been found to be abnormal in the urinary microbiome in connection with bladder cancer (Hussein et al., 2021). It also has been included in a metagenomic panel screening for the diagnosis of ovarian cancer (Kim et al., 2020) and associated with antibiotic perturbation leading to accelerated tumor growth in breast cancer (Kirkup et al., 2019). Interestingly, Cryocola has been found to be increasingly abundant after *H. pylori* eradication in gastric cancer cells (Figueiredo and Castaño-Rodŕıguez, 2020) which may point out to second order competition effects. Dactylosporangium produces molecules such as macrolides that disrupt the mitochondrial membrane potentials in colorectal cancer cells HCT116 and HT29 (Tan et al., 2018) and belong to a class of microorganisms that are being considered as source of bioactive metabolites with pharmaceutical interest (Rangseekaew and Pathom-Aree, 2019).

In the case of Pelomonas, it has been recognized as involved in the onset of multifocal atrophic gastritis with intestinal metaplasia, a likely pre-malignant gastric lesion (Yang et al., 2016). It is also abundant in the tumor microenvironment of up to fifty percent of colorectal tumors in one study (Pierce et al., 2018). Pelomonas also has been found as one of the disrupted genera associated with bladder cancer (Liu et al., 2019; Mansour et al., 2020).

Zymomonas have been recognized to play several roles in cancer. Zymomonas' levan is involved in MMP-9 activation and extracellular matrix remodeling and inflammation (Sturzoiu et al., 2011) and also to induce changes in oxidative states leading to antiproliferative and proapoptotic effects in MCF7 breast cancer cells (Queiroz et al., 2017). Similarly, Methylomonas have been found to be involved in the production of toxin genes that are functional drivers in human colorectal cancer (Dutilh et al., 2013) and in the production of azurin, a known cytotoxic factor regulating cell death (Chakrabarty et al., 2008).

It should be noticed, however, that confirmation studies, in particular functional intervention assays, are needed to establish more clearly the actual role of microbiome dysbiosis in connection with the onset and development of human malignancies in general and specially colon cancer.

### Biological Functionality Associated to the Microbiome Changes With Progression

The concerted study of gene-microbial interactions is still at its infancy. It results challenging thus to ascertain or even hypothesize on the role that microbial communities play in the already complex and incomplete panorama of biomolecular interactions inside human cells and tissues. In order to advance, if just a little, in our understanding of how microorganisms and their joint metabolic fluxes and ecological interactions influence the molecular and cellular composition and functions, we have resorted to analyse the *gene-microorganism co-expression* networks. By looking at the known molecular players (genes) that present strong statistical dependencies with specific microbial species we may start by assigning those (via *guilt-by-association* schemes) a putative functional role in human (in this case, tumor) biology.

Gene enrichment analysis was used to indirectly *probe* associations with the microbiome by looking at the gene-neighborhood of highly connected microorganisms in early and late stage colorectal cancer bipartite networks. Gene Ontology Biological Processes (BP) and Molecular Function (MF) branches were considered as target databases for the statistical over-representation enrichment analysis as presented in Figures 6, 7 for BP, and Figures 9, 10 for MF in early/late colorectal tumor networks, respectively.

As presented in results, we were able to find functional differences between the early and late stage gene-microbiome co-expression programmes. A number of statistical significant processes and molecular functions are presented in the heatmaps in Figures 5, 6, 8, 9. To present a summary of these findings, we used natural language processing tools on tokenized versions of the enrichment tables. Figures 8, 11 present Venn diagrams depicting highly mentioned tokens. We can see that in the case of BP (Figure 8), early stage networks are enriched for terms related to proliferation and cell growth, including structural elements and synthesis of biomaterials, whereas late stage is characterized by terms related to signaling and transport processes. Biochemical and physical regulation mechanisms are present in processes at the intersection of both networks.

Following a similar approach, tokens related to molecular functions associated with early and late stage colorectal cancer are presented in Figure 11. As in the case of biological processes, molecular functions associated with early tumors are related with structural features, late stage contains terms related to signaling and metabolic interactions, whereas the only molecular functions at the intersection of stages are related to binding.

By integrating these results some preliminary ideas may be drawn: first of all, it is becoming possible to analyse (albeit still in a somehow rudimentary way) the combined effect that the microbiome plays in conjunction with human tumor cells in the onset, establishment and development of colorectal cancer. These initial analyses, reveal differences in the functional features of the gene-microbiome bipartite co-expression networks, as inferred from probabilistic modeling of high-throughput genomic and transcriptomic experiments in large datasets. These differences, when supplemented with statistical enrichment analyses point out to a plausible scenario in which early stage colon cancer presents features related to the establishment of distinctive physical structures in the cells, that start to couple with biomolecular interactions at the cellular level, whereas advanced stages present an image of more complex signaling and metabolic processes occurring as the tumor keeps evolving to more advanced, malignant stages.

### Scope and Limitations

In this work we identify changes in the co-expression/co-presence network connectivity found between colon cancer microbiome and its gene expression as the disease progresses. This type of studies are admittedly at their preliminary stages, but the integrative view they aim to provide seems promissory toward a better understanding of complex disease phenotypes. It is relevant, however, to acknowledge some limitations and assumptions of our current approach, in order to properly contextualize our findings and convey a balanced message.

One worth-mentioning constraint that may restrict the scope of our assertions is the following: Our work is based on experimental data coming from the TCGA colon cancer cohort. The volume of this cohort, as well as the availability of proper, well-curated, clinical metadata, makes it suitable for our (high-throughput, probabilistic-based) analyses. Furthermore, the open microbiome quantification strategy and the resulting data from Poore et al. (2020) allowed for a (relatively) high-confident network reconstruction. This is, however, the only cohort for which such suitable data is available, thus limiting our ability to replicate our findings in an independent cohort. While the sample size is adequate for probabilistic network reconstruction purposes, it can only capture as much of the microbiome heterogeneity as what was captured by the original authors. On a related topic, since access to the TCGA raw data required for the microbiome quantification data described in Poore et al. (2020) is controlled, we must rely on the quantification strategy as performed by the original authors—which is in turn influenced by sequencing depth and wet-lab procedure constraints from the original work.

Aside from these specific issues, some additional, general limitations should also be mentioned: although the methods used both in our work and in Poore et al. (2020) and even those in the TCGA original approach are all in the state of the art, there are still challenges. Even though the TCGA data has both, excellent depth and high quality sequencing, it was not intended as a metagenomic sequencing assay. Also, even the best metagenomic approaches rely on currently incomplete annotations. Pre-processing stages to consider multi-omic approaches, including metagenomic data are being developed so, these may not be as optimized and standardized as it will be desirable.

In spite of these clear limitations, we are convinced of the value of approaches such as the one presented here to start trying to answer these questions from an integrative data-centered view.

## Conclusions

The progression of colon cancer involves changes in the interactions between cancer tissue and microbiome. In this work, we integrated microbiome quantification data with gene expression data using network models. These models describe the aforementioned changes in this interactions. We found that indeed, the set of microorganisms with a higher connectivity with host genes changes from the early to the late stages of colon cancer. Furthermore, reorganization is accompanied by changes in the associated set of biological functions, showing physiological adaptations associated to the tumor-microbiome relationships. To better understand and validate this findings, future experimental work is needed to properly characterize the mechanisms through which the microbiome may be mediating the observed tumor adaptations.

## Data Availability Statement

The original contributions presented in the study are included in the article/Supplementary Material, further inquiries can be directed to the corresponding author/s.

## Author Contributions

IU-N organized data, performed calculations, and analyzed the data. EH-L co-designed the study, contributed to the methodological approach, analyzed data, discussed results, reviewed the manuscript, and co-supervised the project. GdA-J envisioned the project, devised the methodological strategy, developed code, performed calculations, analyzed data, discuss results, drafted the manuscript, and co-supervised the project. All authors read and approved the final manuscript.

## Conflict of Interest

The authors declare that the research was conducted in the absence of any commercial or financial relationships that could be construed as a potential conflict of interest.
