# Supplementary material for: Gene-Microbiome Co-expression Networks in Colon Cancer
Source: Front Genet. 2021 Feb 15;12:617505. doi: 10.3389/fgene.2021.617505 (PMC7917223; doi:10.3389/fgene.2021.617505)
Supplement: Supplementary file 2 [file Data_Sheet_2.PDF]

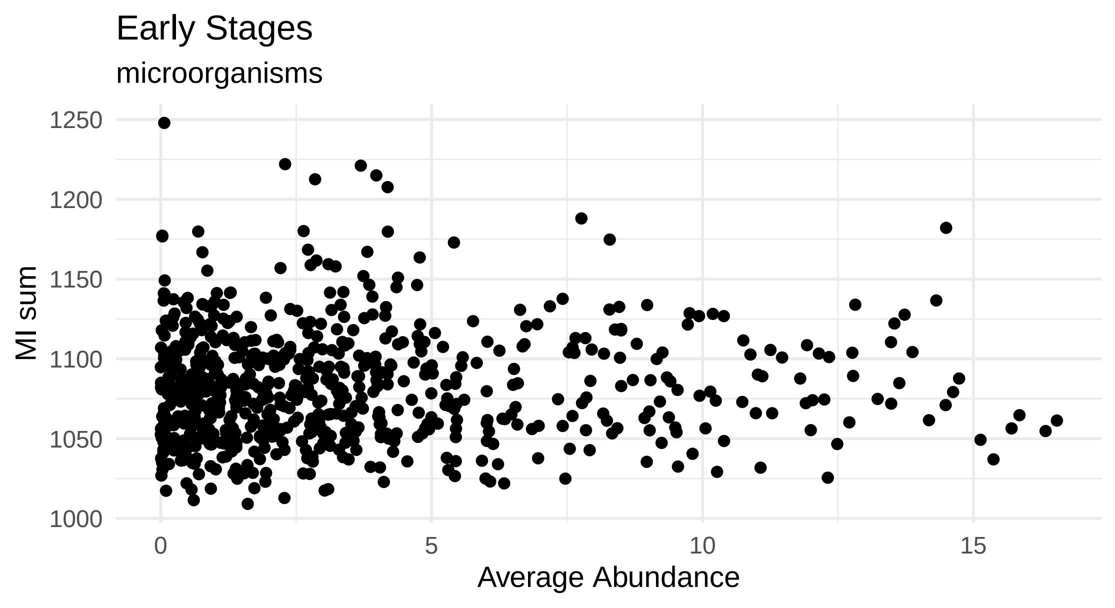

Early Stages
microorganisms
MI sum
1250
1200
1150
1100
1050
1000
0
5
10
15
Average Abundance

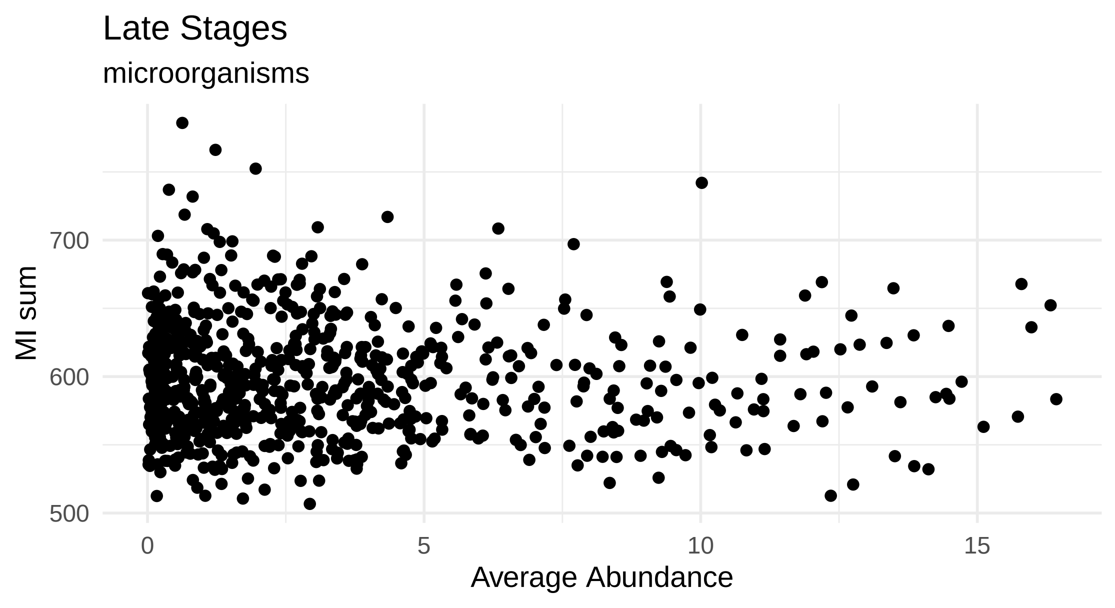

Late Stages
microorganisms
MI sum
700
600
500
0
5
10
15
Average Abundance

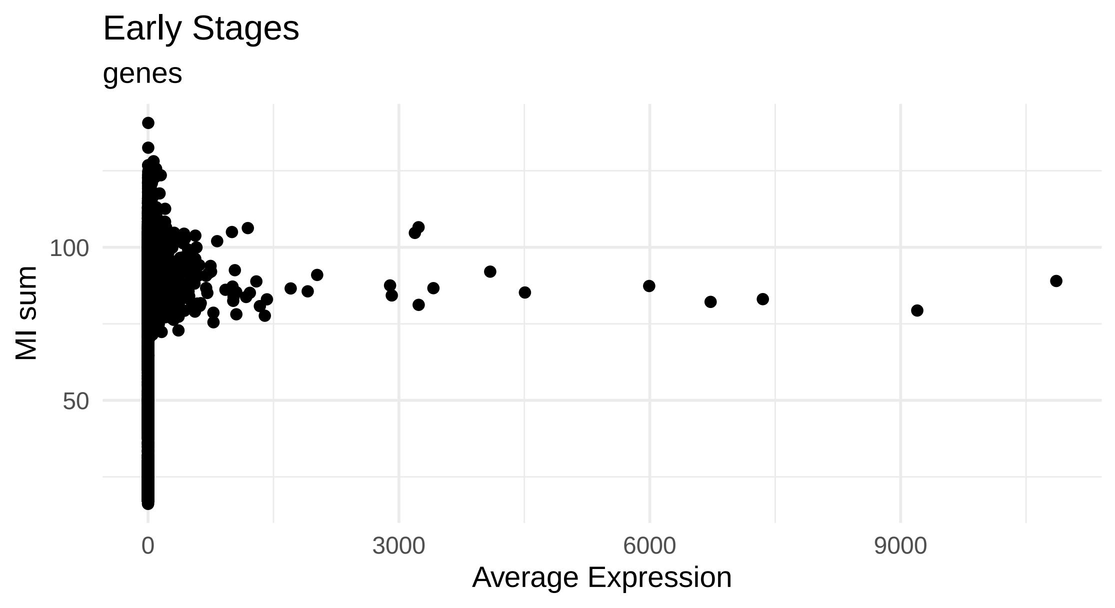
Early Stages
genes
MI sum
100
50
0
3000
6000
9000
Average Expression

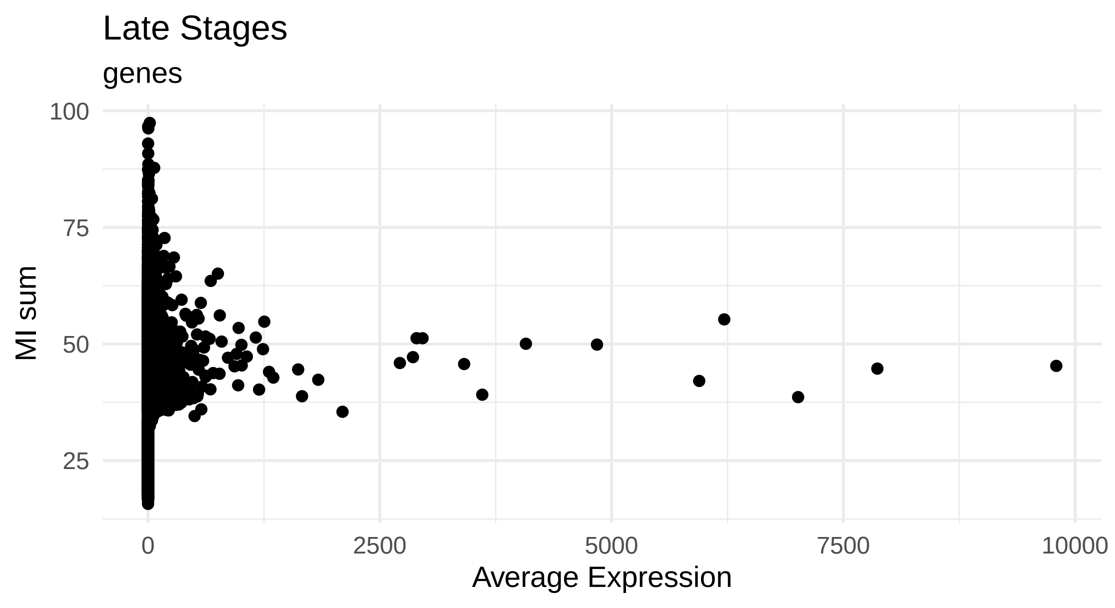
Late Stages
genes
MI sum
Average Expression
100
75
50
25
0
2500
5000
7500
10000

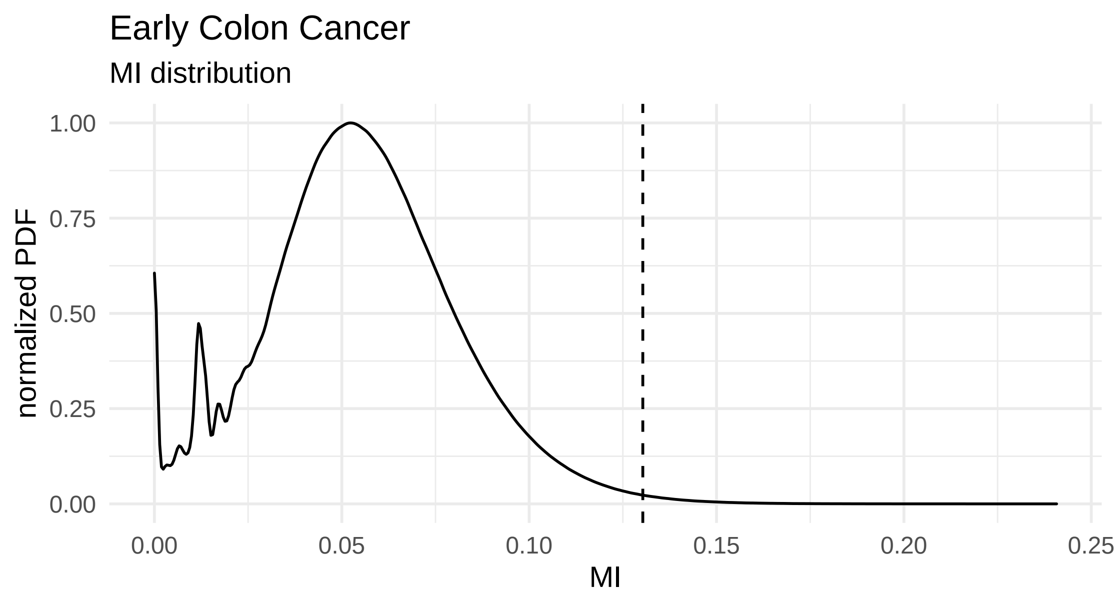
Early Colon Cancer
MI distribution
normalized PDF
1.00
0.75
0.50
0.25
0.00
0.00
0.05
0.10
0.15
0.20
0.25
MI

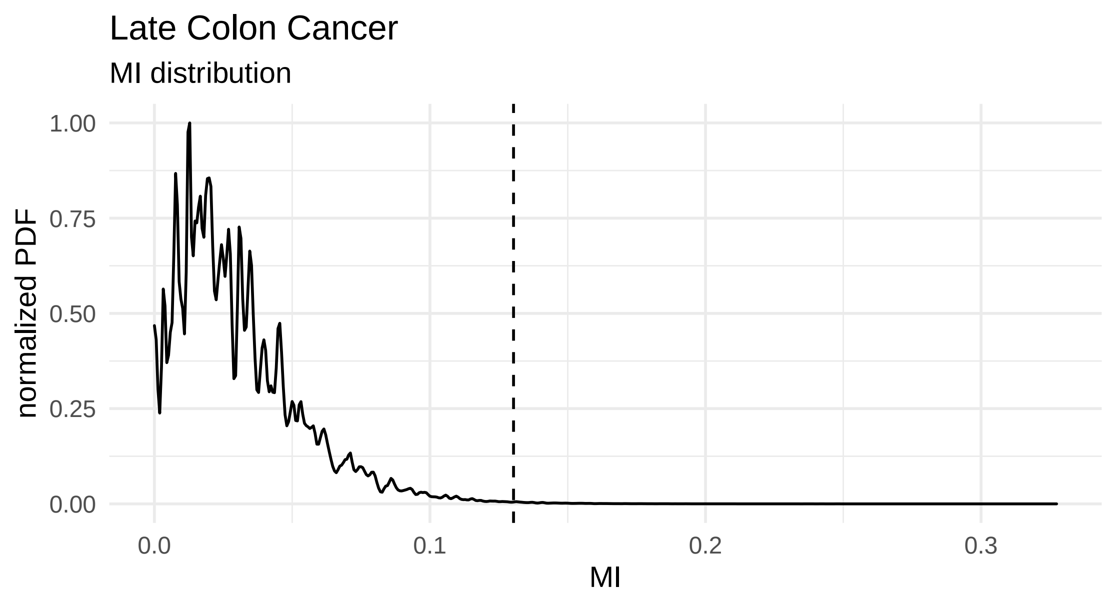
Late Colon Cancer
MI distribution
normalized PDF
1.00
0.75
0.50
0.25
0.00
0.0
0.1
0.2
0.3
MI
